# A case series of FDG PET scan: hypometabolic lesions that matter in oncology

**DOI:** 10.1259/bjrcr.20220144

**Published:** 2023-02-20

**Authors:** Tak Kwong Chan, Koon Kiu Ng, Boom Ting Kung, Ting Kun Au Yong

**Affiliations:** 1 Nuclear Medicine Unit, Queen Elizabeth Hospital, Hong Kong, China

## Abstract

Interpretation of FDG PET images in oncology patients is in general a visual exercise of search for focal increased uptake (hypermetabolism). However, in some cases, hypometabolism (focal decreased uptake) can matter as much as hypermetabolism. We report three cases of FDG PET studies for oncological indications. All of them showed focal hypometabolic lesions suspicious of metastases. The diagnoses were then supported either by histological proof and/or follow-up imaging studies. The importance of being alert to both focal hypermetabolism and focal hypometabolism when interpreting FDG PET images is underscored.

## Background

Positron emission tomography (PET) is a nuclear functional imaging that can demonstrate a wide variety of physiological and pathological processes at molecular levels by means of showing the distribution of injected radiotracers. Computed tomography (CT) is now usually performed simultaneously with PET to provide anatomical details for attenuation correction and anatomical localisation. Fluorine-18 fluorodeoxyglucose (FDG) is the most widely used radiotracer for PET. It is a glucose analogue and its uptake intensity in target tissues can reflect regional glucose consumption. Many cancer cells have increased cellular glucose transporters and hexokinase activity to support cell growth compared with normal cells and would show focal increased FDG uptake.^1^ This renders FDG PET the non-invasive imaging of choice for detection, staging, and therapy response assessment in many oncological diseases.^2^ In general, FDG PET is interpreted by visual search for abnormal focal increased tracer uptake. However, hypometabolic lesions sometimes can be of huge clinical significance. We report three oncological cases to illustrate the importance not to miss lesions of abnormal reduced FDG uptake.

## Case 1

**Figure 1.**
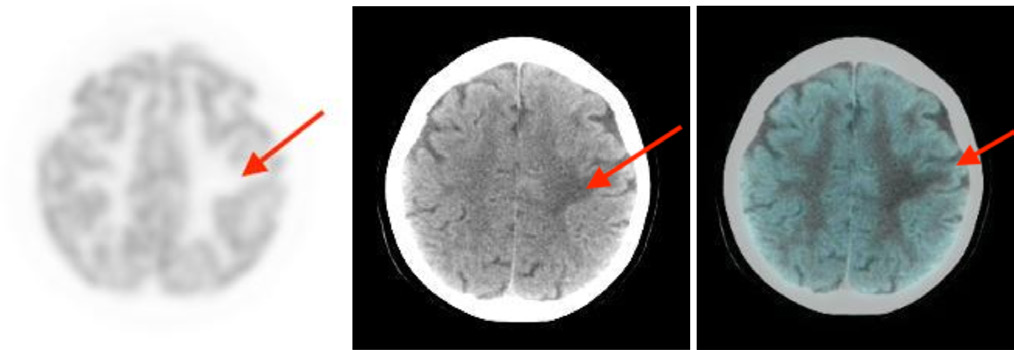
FDG PET (left) showed asymmetrical hypometabolism in left parietal lobe (red arrow) associated with peri-focal oedema on CT (middle).

**Figure 2.**
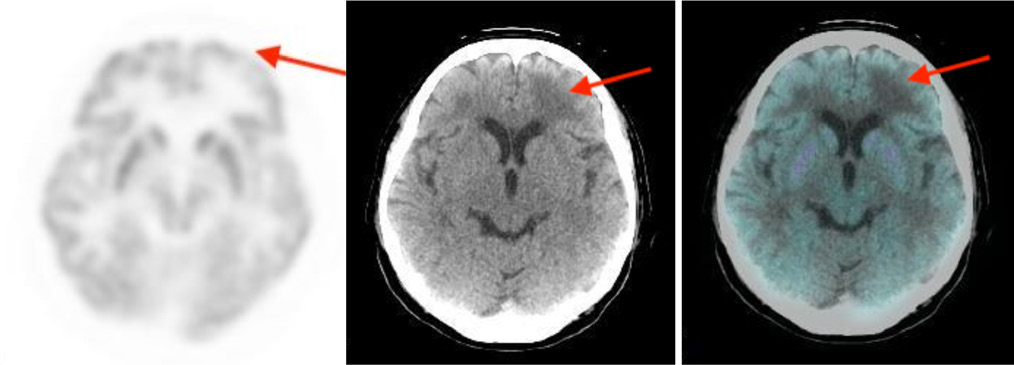
FDG PET (left) showed asymmetrical hypometabolism in left frontal lobe (red arrow) associated with peri-focal oedema on CT (middle).

**Figure 3.**
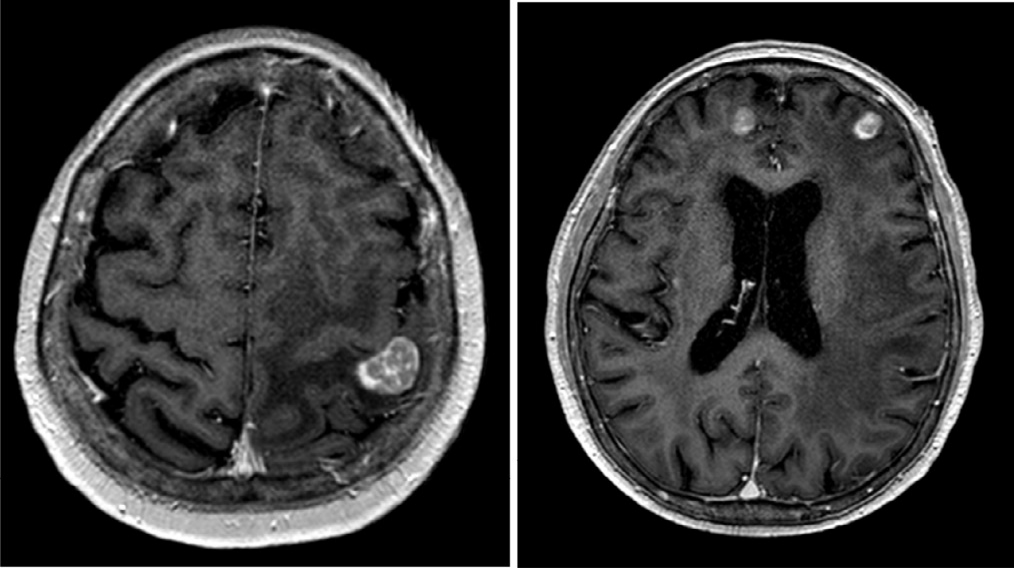
MRI brain showed contrast enhancing brain metastases in left frontal and parietal lobes. There was also another metastasis in right frontal lobe.

A 75-year-old female had history of right mastectomy for invasive ductal carcinoma. She then received adjuvant radiotherapy and hormonal therapy. A year later, she had adenocarcinoma in upper lobe of right lung with nodal spread to left supraclavicular fossa, which was treated with gefitinib. In the FDG PET follow-up reassessment, there was a new hypermetabolic focus in spinal region of C5 vertebral level, suspicious of intramedullary or leptomeningeal metastasis. Radiotherapy was then given. A follow-up FDG PET scan showed resolution of the hypermetabolism in C5 level. But there was new subtle asymmetrical hypometabolism in left frontal and parietal lobes of the brain (Figures 1 and 2). Corresponding CT showed perifocal oedema in left frontal lobe and parietal lobes with no apparent focal lesion. The findings raised suspicion of new brain metastases. Magnetic resonance imaging (MRI) subsequently performed affirmed the suspicion by showing contrast enhancing brain lesions in left frontal and parietal lobes (Figure 3). There were also a few other smaller brain metastases involving bilateral cerebral and cerebellar hemispheres.

## Case 2

**Figure 4.**
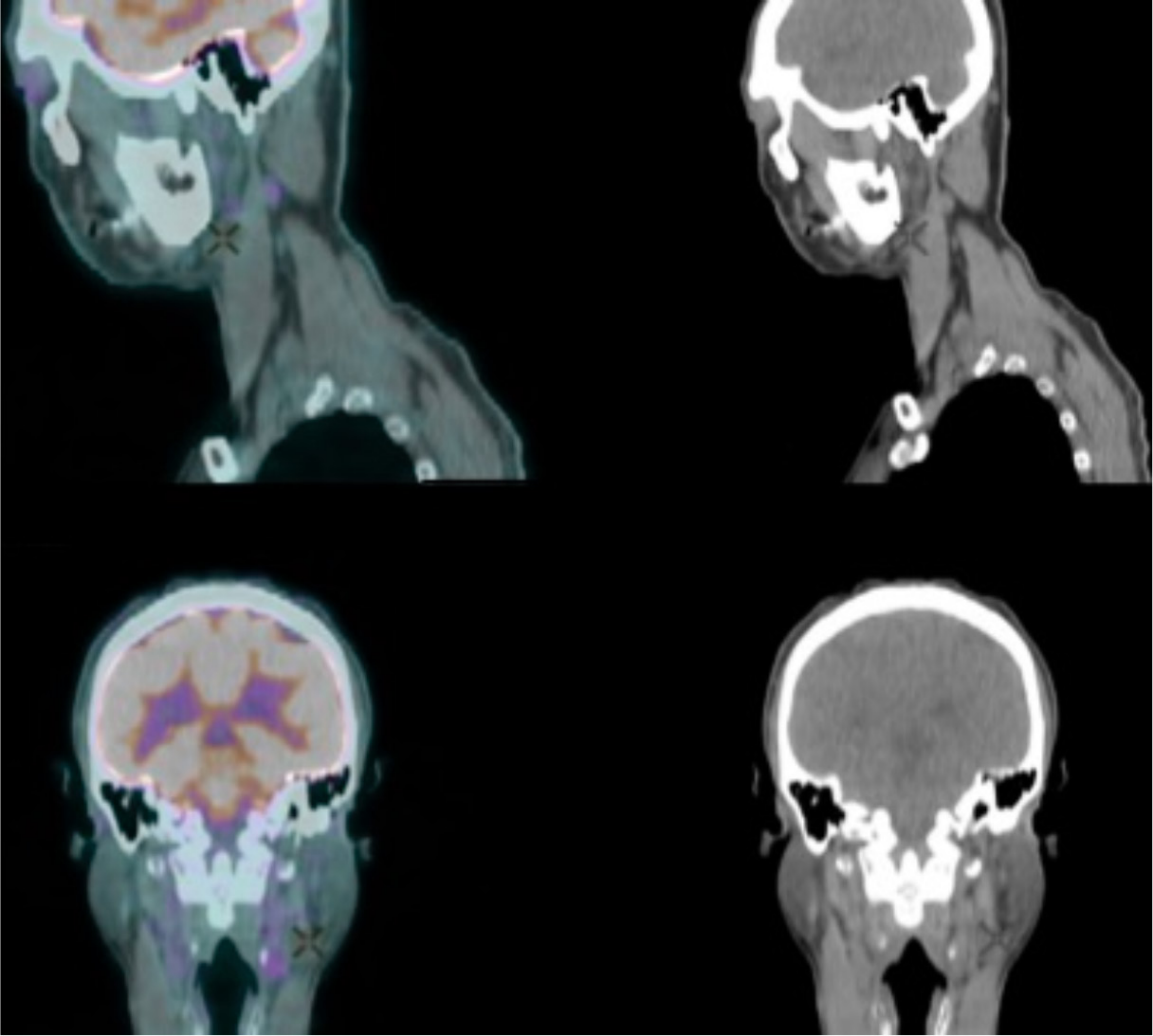
FDG PET shows hypometabolism in the concerned left upper neck lesion (cross).

**Figure 5.**
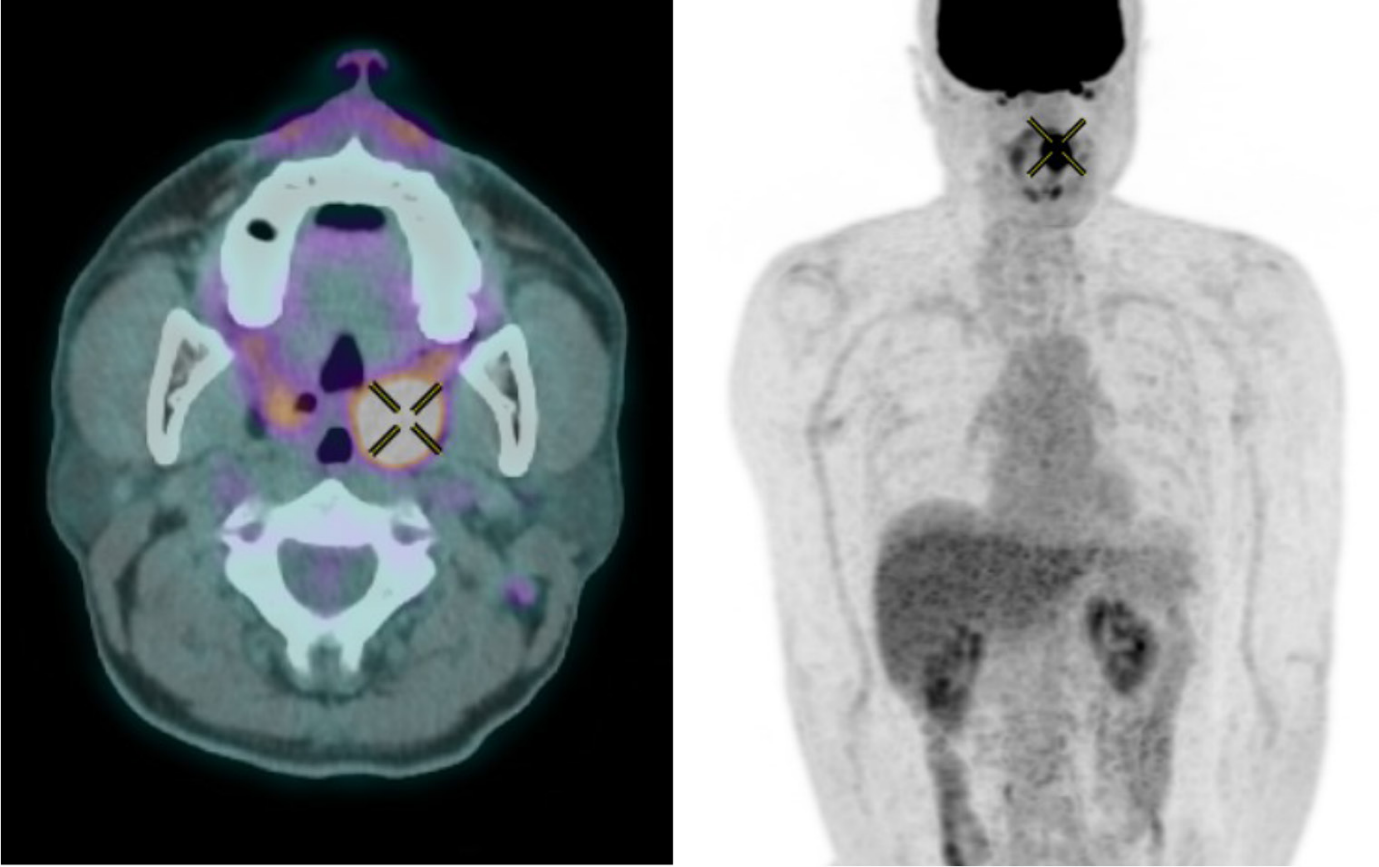
FDG PET showed hypermetabolism in left tonsillar region highly suspicious of malignancy, which proved to be squamous cell carcinoma.

A 52-year-old male complained of a left upper neck mass. Fine needle aspiration cytology showed atypical cystic lesion. MRI was subsequently done showing a well circumscribed cystic lesion suspected of a branchial cleft cyst. FDG PET showed hypometabolism in the concerned left upper neck lesion (Figure 4). There were an intensely hypermetabolic left tonsillar lesion highly worrisome of malignancy (Figure 5) and a few other mildly FDG-avid left upper jugular and supraclavicular shotty lymph nodes. Tonsillectomy was performed. Histology confirmed squamous cell carcinoma. Intra-operative fine needle aspiration cytology of the concerned left upper neck lesion showed scanty cells suspicious of metastatic squamous cell carcinoma.

## Case 3

**Figure 6.**
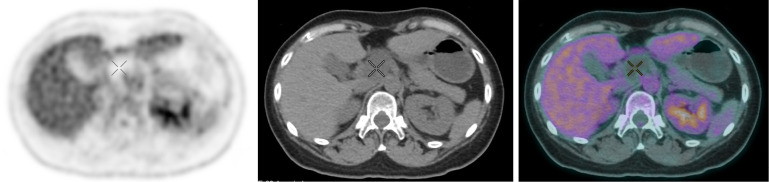
FDG PET (left) showed hypometabolism in the concerned lesion abutting the caudate lobe of liver (cross).

**Figure 7.**
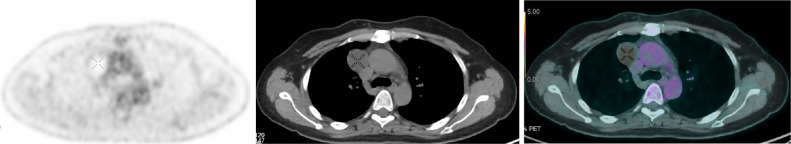
FDG PET (left) showed several other hypometabolic thoracic nodes (cross).

A 45-year-old female with clear cell carcinoma of ovary had total abdominal hysterectomy, bilateral salpingo-oophorectomy and bilateral pelvic nodal dissection. She then received adjuvant chemotherapy. A follow-up CT showed a few non-specific hepatic hypodense lesions. MRI was subsequently performed, showing a 4-cm fluid-filled lesion abutting the caudate lobe of liver. FDG PET performed to characterise the lesion showed hypometabolism (Figure 6). There were also multiple nodal lesions in superior mediastinum (Figure 7), right infraclavicular region and left internal mammary station, all showing hypometabolism. Overall, FDG PET findings raised suspicion of multiple cystic/necrotic nodal metastases. Bronchoscopic ultrasound-guided fine needle aspiration cytology of one of the mediastinal nodes showed carcinoma cells compatible with metastases from female genital tract cancer.

## Discussion

The most common primary tumours metastasising to the brain are lung, breast and melanoma.^3^ About 80% of brain metastases are found in the cerebral hemispheres, 15% in the cerebellum and 5% in the brainstem.^4^ Contrast CT is widely used for the detection of brain metastases for its accessibility speed and low cost. Contrast MRI is currently the imaging of choice.

Sensitivity of FDG PET to detect brain metastases was considered to be limited by false-negative cases due to masking by intense physiological activity in the brain. Some lesions may be too small to be detected and some lesions may show normal or reduced FDG uptake. A meta-analysis of five studies showed a sensitivity of only 21% for FDG PET to detect brain metastases from lung cancer. However, some included studies performed FDG PET only without CT and none disclosed whether hypometabolic lesions were counted towards a positive scan.^5^

In a previous review of 71 metastatic brain lesions, when compared to contralateral normal brain FDG uptake, 41 lesions (57.7%) showed less intense FDG uptake (hypometabolic), three lesions (4.2%) showed more intense FDG uptake (hypermetabolic), 27 lesions (38.0%) showed similar FDG uptake. On per-patient basis, FDG PET has a sensitivity of 79% for detection of brain metastases.^6^ Another study recruited 1108 lung cancer patients with 66 cases of brain metastases. Focal increased or decreased FDG uptake were considered as positive. It showed a sensitivity of 72% and specificity of 100% of FDG PET for detection of brain metastases.^7^ Notably if we include the both hypermetabolic and hypometabolic lesions as positive lesions, it is likely that sensitivity can be significantly increased. Hypometabolism may result from perifocal vasogenic oedema, cystic, necrotic, calcific or haemorrhagic components or inherent properties of tumour cells.

However, it should be borne in mind that for those patients who have undergone radiation therapy for brain metastases, either radiation necrosis or recurrent metastases can exhibit hypometabolism.^8^ Patients with previous cerebrovascular accident, epilepsy, cerebral trauma, cerebral infection, other brain neoplasm or neurodegenerative diseases may also have pre-existing asymmetrical hypometabolism. Granted the limited diagnostic performance of brain FDG PET, it can provide valuable complementary information if sufficient attention is paid to detect asymmetrical cerebral and cerebellar hypermetabolism and hypometabolism and make reference to relevant clinical history regarding previous irradiation or other pre-existing neurological disease.

So far as nodal metastases are concerned, FDG PET has proven value of nodal staging in a number of different cancers, such as lung, melanoma, sarcoma, head and neck cancer.^2^ While it is mostly utilised in visualisation of focal hypermetabolic nodes, it is also known that necrotic or cystic lymph nodes may not always contain sufficient metabolically active tissue to show FDG uptake, even although the primary malignancy does show increased FDG uptake.^9^ Cystic change in metastatic lymph nodes occurs in certain types of tumours and most commonly arise from squamous cell carcinoma in head and neck region.^10^ Other reported primary tumours known to have cystic nodal metastases are thyroid papillary carcinoma, serous papillary carcinoma of the ovary/endometrium and malignant melanoma.^11^ For these cancers in particular, nuclear medicine physicians should pay attention to metastatic nodal lesions that may exhibit hypometabolism due to cystic change.

## Conclusions

It is important to note that hypometabolism can matter as much as hypermetabolism on FDG PET scans. The findings on FDG PET in the reported cases should be treated as true positive rather than false negative because focal hypometabolism should be considered a positive finding in proper clinical context. When interpreting FDG PET images for these oncological indications, one should be equally alert to both focal hypermetabolism and focal hypometabolism.

## Learning points

1. Brain metastases may show asymmetrical hypometabolism on FDG PET due to perifocal vasogenic oedema, cystic, necrotic, calcific or haemorrhagic components and/or inherent properties of tumour cells.
2. Necrotic or cystic metastatic nodes arising from head and neck squamous cell carcinoma, thyroid papillary carcinoma, serous papillary carcinoma of the ovary/endometrium and malignant melanoma may show hypometabolism on FDG PET.
3. When interpreting FDG PET images for oncological indications, one must be alert to both focal hypermetabolism and focal hypometabolism.
